# Epidemiological assessment of the factors associated with antimicrobial use in French free-range broilers

**DOI:** 10.1186/s12917-019-1970-1

**Published:** 2019-06-28

**Authors:** Cécile J. M. Adam, Nicolas Fortané, Alexandra Coviglio, Léa Delesalle, Christian Ducrot, Mathilde C. Paul

**Affiliations:** 1 ENVT, INRA, UMR 1225 IHAP, 23 chemin des Capelles, 31076 Toulouse, France; 2 EPIA, INRA, VetAgroSup, 63122 Saint-Genès-Champanelle, France; 3 UMR IRISSO, CNRS, INRA, Paris-Dauphine, PSL, Place du Maréchal de Lattre de Tassigny, 75775 Paris cedex 16, France

**Keywords:** Risk factor, Antibiotic, Pharmaco-epidemiology, Poultry, Case-control study

## Abstract

**Background:**

Although the poultry sector accounts for a major portion of global antimicrobial consumption, few studies have explored the factors which influence antimicrobial use (AMU) in poultry farms in Europe. We performed a matched case-control study in traditional free-range broiler farms in France during 2016 to evaluate the effect of technical factors and farmers’ perceptions of health problems on the probability of AMU. In total, 52 cases (defined as flocks treated with antimicrobials when chickens were between 1 and 42 days old), were included. Another 208 controls (untreated flocks the same ages as the case flocks), were randomly selected and paired with a matching case (same farmer organization and placement date). On-farm questionnaires were administered. Multivariable logistic regression modeling was conducted; seven variables were significant in the final model.

**Results:**

Two factors were associated with a lower probability of AMU: the use of chicken paper topped with starter feed (OR = 0.3; 95% CI = [0.1; 0.9]) and the use of herbal drugs as a prophylaxis (OR = 0.1; 95% CI = [0.01; 0.5]). A higher probability of AMU was associated with farmers perceiving the cumulative mortality of chicks between 1 and 10 days old as normal (OR = 10.1; 95% CI = [1.7; 59]) or high (OR = 58.7; 95% CI = [9.6; 372.3]). A higher probability of AMU also was associated with farmers detecting a health problem (OR = 12.5, 95% CI = [4.2; 36.9]) and phone calls between farmers and their technicians (OR = 5.9; 95% CI = [2.3; 14.8]) when chicks are between 11 to 42 days old. Two additional factors (litter thickness and cleaning/disinfecting) were significant and highlighted the importance of technical factors such as biosecurity.

**Conclusions:**

Our results suggest that to reduce AMU, technical training should be provided to farmers to improve how farms are monitored and to reinforce preventive health measures. Training also should address how farmers assess warning criteria like daily mortality rates, which when overestimated often lead to antimicrobial treatment.

**Electronic supplementary material:**

The online version of this article (10.1186/s12917-019-1970-1) contains supplementary material, which is available to authorized users.

## Background

Antimicrobial resistance (AMR) is a major global public health issue with a significant impact on national health budgets. AMR is responsible for approximately 700,000 deaths each year, and is expected to cause one death every three seconds worldwide by 2050 [1]. The development of bacterial resistance to one or several antimicrobials is associated with the overuse of antimicrobials in human and veterinary medicine [2]. However, it is difficult to establish direct causality, and the risk of AMR in livestock affecting humans is difficult to quantify [3]. Global consumption of antimicrobials is expected to increase by 67% between 2010 and 2030 and it could be challenging to limit antimicrobial use (AMU) in food-producing animals [4]. Species reared in intensive production systems such as poultry and swine have been identified as major drivers for antimicrobial use and subsequent development of antimicrobial resistance [4].

Veterinarians and poultry professionals (farmers and technical advisors) should emphasize the prudent use of antimicrobials. Antibiotics should be strictly used only when required and care should be taken not to endanger animal health and/or welfare [5]. To limit on-farm use of antimicrobials, the factors driving this use must be identified, yet only a limited number of articles have addressed this topic to date. Most research on this topic has focused on cattle [6–8] and pigs [9–12]. In pig production systems, epidemiological studies have highlighted the impact of various farm characteristics on the use of antimicrobials, including farm density in an area, farm type, herd size, and biosecurity measures [9–12]. Surprisingly, there is a paucity of articles on the factors associated with AMU in poultry in Europe [13]. Recent studies have made it possible to precisely quantify exposure to antimicrobials in Belgian [14] and Canadian [15] broilers using various metrics, but these works did not investigate the factors associated with between-flock variations in AMU. With regard to the factors driving AMU, Chauvin et al. [16] showed that farmers’ expectations played a key role in the prescription of antimicrobials by veterinarians. Protective practices, such as administration of competitive exclusion flora and compliance with biosecurity rules (changing clothes and shoes before entering the facilities), were shown to be associated with lower AMU. Hughes et al. [17] investigated the indications for therapeutic and preventive use of antibiotics in broilers, as well as the effect of farm management practices. They found that the use of competitive exclusion products, the use of antibiotic growth promoters and controlled feeding regimens were all associated with a reduced risk of the use of antibiotics for preventive purposes. However, considerable changes in the regulatory framework and antimicrobial prescription practices have occurred since the latter two studies were conducted (in particular regarding the use of antibiotics for preventive purposes); the results, therefore, should not be extrapolated to current poultry production. Updated knowledge on risk factors associated with AMU in poultry in Europe is thus needed. In addition to the conventional prevention strategies (poultry farm equipment, biosecurity and prophylaxis) investigated in the studies mentioned above, farmers and health advisors are increasingly interested in the use of alternative prevention strategies, including vaccines, prebiotics, probiotics, and herbal drugs, to improve the production performance and health status of livestock. This recent trend, which remains poorly documented, should also be considered in epidemiological research.

Wauters and Rojo-Gimeno [18] argue that veterinary epidemiology should develop socio-psychological approaches focusing on how human behavior affects the causes, spread, prevention and control of animal health problems to complement analyses of farm characteristics and farming practices. Previous studies have highlighted the importance of considering factors such as the attitudes, risk perception [12], and profiles of antimicrobial users [9]. Concerning the management of disease situations, Alarcon et al. [19] have stressed the importance of considering farmers’ perceptions to better understand their decisions. Despite those converging conclusions, few epidemiological studies have investigated the collective impact of a wide range of technical, sociologic and economic factors on AMU. The possibility that farmers and veterinarians do not perceive a given health situation in the same way also has rarely been explored.

The present case-control study aimed to quantify the impact on AMU of various factors related to daily farm management practices, including farmer perception of animal health and the use of alternatives to antibiotics for the prevention of diseases in poultry. This study was performed in French traditional free-range broiler farms during the indoor production period. Under French regulations, traditional free-range systems must raise slow-growing strains of chickens, and day old broilers are raised indoors until day 42. Given that 75% of antimicrobial treatments are administered during the first 42 days of the broiler production system, the current study focused on the indoor production period (days 1 to 42).

## Results

### Participation

Of the nine FOs initially recruited for the study, one withdrew early on, and one did not report any AMU. Consequently, seven FOs ultimately were involved in the survey.

Out of the 315 farmers contacted, 16 (5%) were unreachable and 23 (7%) refused to participate. The refusals were mostly motivated by a lack of time (17/23) or lack of motivation to answer another questionnaire (2/23). Four farmers did not provide any explanation for their refusal. Refusals and unreachable farmers were almost exclusively control flocks. Seven farmers were excluded after it was realized that their flock did not match the case or control definition.

Among the 273 flocks visited, 19 (7%) had been wrongly classified: 14 flocks selected as controls were identified as cases after the on-farm visit, and five flocks initially considered as cases did not use antimicrobials and were re-classified as controls.

After excluding the flocks with missing information, the final sample contained 260 flocks (52 cases and 208 controls) for statistical analysis with a median number of 30 farms per FO. The validity of the data entry was assessed on 15 control-questionnaires and 15 case-questionnaires and was determined to be correct.

### Sample description

**Fig. 1:**
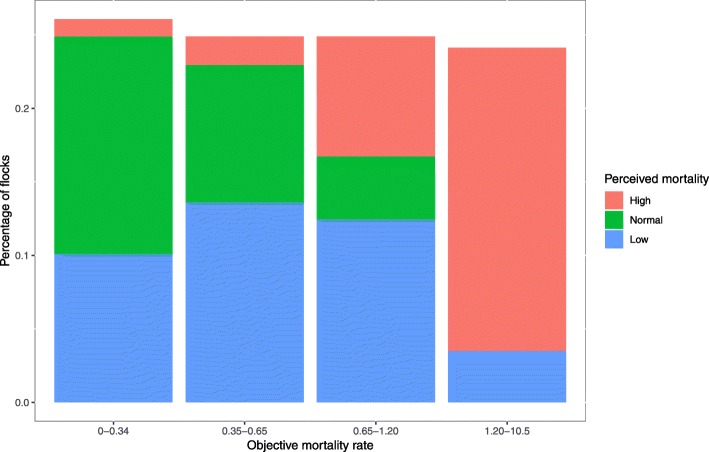
Distribution of the crude objective 10-day mortality rate (%) and farmer perception of the 10-day mortality rate in a case-control study of antimicrobial use in French traditional free-range broiler farms (260 flocks), 2016

The median age of the farmers was 49 years (Interquartile Range IQR = 13). The median total surface area of the poultry houses was 800 square meters (IQR = 814). Further description of the sample can be found in Table 1. The crude objective 10-day mortality rate was 0.63%, with an interquartile range of 0.83%. For 74 flocks, the 10-day mortality rate was judged by the farmer as “low”, for 103 flocks it was judged “normal”, and for 83 it was judged “high”. The correlation between these two variables can be assessed in Fig. 1.

**Table 1:** Description of the sample of 260 flocks, in a case-control study performed in 2016 on antimicrobial use on traditional French free-range broiler farms

	Category	Total	%
Gender	Women	73	28.1
	Men	187	71.9
Contribution of free-range broiler breeding to total farm income in 2015	Under 25%	55	21.2
	25–50%	82	31.5
	50–75%	53	20.4
	75–100%	37	14.2
	100%	29	11.2
	Missing data	4	1.5
Crop production in 2016	Yes	236	90.8
	No	24	9.2
Other breeding production in 2016	Yes	128	49.2
	No	132	50.8
Conventional broiler production in 2015	Yes	28	10.8
	No	232	89.2
Anseriformes bred in 2015	Yes	30	11.5
	No	230	88.5

### Description of the antimicrobial treatments

In the 52 case flocks, a total of 61 antimicrobial treatment events were noted. Six of the treatments were administered as a preventive measure and 55 as a therapeutic measure. Of the 55 therapeutic treatments, five flocks received two antimicrobial treatments for the same health problem according to the farmer. One flock received three antimicrobial treatments to deal with three different health problems according to the farmer.

**Fig. 2:**
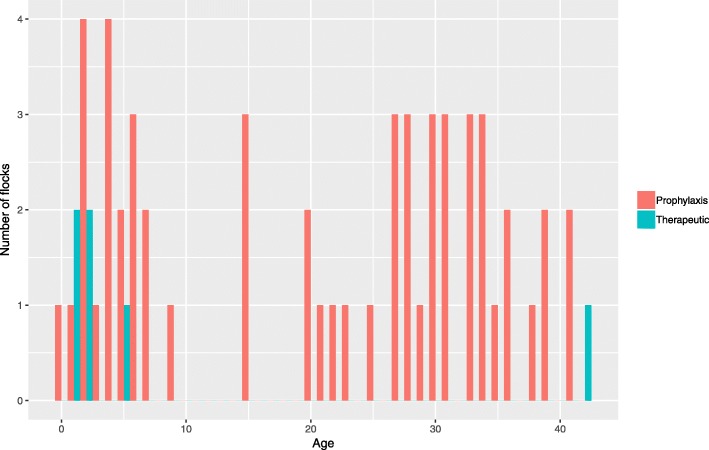
Age and purpose of antimicrobials administration for the case flocks (n = 52) in French free-range broilers (*n* = 61 antimicrobial administrations), 2016

The median age of the broilers for the 61 antimicrobials administered was 22 days, with an interquartile range of 26 days. In total, 18 treatments were made between 1 and 5 days (Fig. 2). Two treatment peaks were observed: during the five first days and between 27 and 33 days of age. Five antimicrobial treatments out of the six administered as a prophylaxis were made before day 5.

**Fig. 3:**
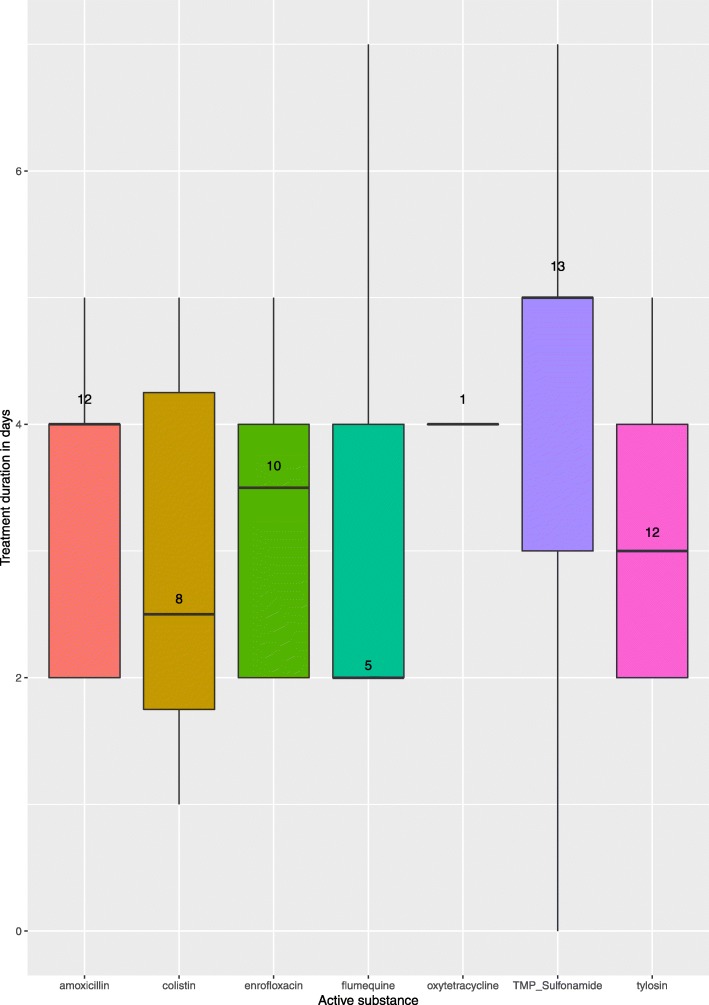
Distribution of the duration of antimicrobial treatments for each active substance (n = 61) in French free-range broiler flocks (*n* = 52), 2016

The four main active substances used were sulfonamides, amoxicillin, tylosin, and enrofloxacin (Fig. 3).

Most of the health problems associated with the therapeutic uses were abnormal mortality (identified by farmers 51 times) and digestive disorders (identified 13 times). Among the 55 flocks treated with therapeutic antimicrobials, there were a total of 41 autopsies performed by veterinarians or technicians and 16 sensitivity tests performed (for which only six reports were found on farm and photographed).

The median duration of antimicrobial treatment was four days, with an interquartile range of two days. Wider variation was observed for flumequine and TMP sulfonamides (Fig. 3).

### Description of the health problems

**Fig. 4:**
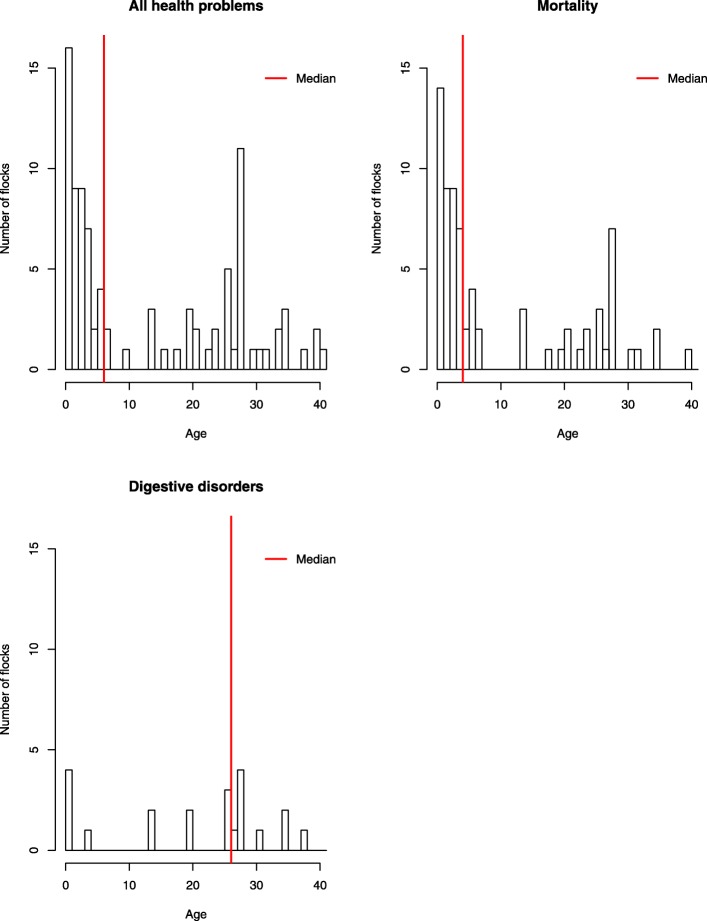
Distribution and mean of the age at the beginning of the 92 health events in the 260 French traditional free-range broiler flocks, 2016

Of the 260 flocks studied, 92 had at least one health problem. Of these 92 flocks, 48 were case flocks (receiving antimicrobial treatment) and the remaining 44 were controls (flocks which did not receive any antimicrobial treatment). Farmers could choose several answers to describe the category of health problem which they had identified. The median age of chicks when the health problem occurred was 6 days, with an interquartile range of 25. Abnormal mortality was identified by farmers 72 times, at a median age of 4 days with an interquartile range of 21. Digestive disorders were identified 22 times, and occurred later than abnormal mortality, with a median age of 26 days and an interquartile range of 14 days (Fig. 4).

### Logistic regression model

In total, 48 variables had a *p*-value < 0.25 in the univariable analysis and were thus considered for multivariable analysis. Following the exclusion of correlated explanatory variables, 24 variables were included in the logistic regression model. After the backward selection process, the final model included seven significant variables (Table 2) together with an FO variable that was forced into the model to account for matching [18]. Three variables were associated with a decreased probability of antimicrobial treatment: the use of chicken paper topped with starter feed (OR = 0.29; 95%CI = [0.09; 0.87]), a litter thickness of 10 cm or less (OR = 0.22; 95%CI = [0.06; 0.80]) and the use of herbal drugs as prophylaxis (OR = 0.09; 95% CI = [0.01; 0.49]). Four variables were found significantly associated with an increased probability of treatment: (i) cleaning and disinfection of the concrete perimeter of the poultry house at the previous downtime (OR = 3.43, 95% CI = [1.28; 9.22]), (ii) the farmer’s perception of the mortality between 1 and 10 days of age as “normal” (OR = 10.15; 95%CI = [1.75; 58.97]) or “high” (OR = 58.71, 95% CI = [9.56; 372.26]) in comparison with “low”, (iii) the identification of a health problem by the farmer (OR = 12.47, 95% CI = [4.21; 36.91]) between 11 to 42 days of age, and (iv) a phone call between the farmer and the production technician (OR = 5.87; 95% CI = [2.32; 14.85]) during the same period. This latter variable included all phone calls, regardless of the reason for the call. The model fits well with the observed data (*p*-value = 0.91 of Hosmer Lemeshow test).

**Table 2:** Results of the multivariable regression for the probability of a French traditional free-range broiler flock to receive an antimicrobial treatment (cases, *n* = 52) compared to no antimicrobial treatment (controls, *n* = 208) up to 42 days of age, as adjusted by the farmer organizations, in 2016

Variable	No. flocks	Odds Ratio	95% CI	*p*-value
Use of chicken paper topped with starter feed				
Yes	213	0.29	0.09–0.87	0.027
No	47	Reference		
Use of herbal drugs as a preventive measure				
Yes	37	0.09	0.01–0.49	< 0.01
No	223	Reference		
Thickness of the litter				
10 cm or less	62	0.22	0.06–0.80	0.021
11–15 cm	81	1.19	0.43–3.28	0.730
15 cm or greater	117	Reference		
Cleaning and disinfection of the concrete perimeter of the poultry house at the previous downtime				
Yes	154	3.43	1.28–9.22	0.014
No	106	Reference		
Perception of the mortality rate at 10 days				
High	83	58.71	9.56–372.26	< 0.01
Normal	103	10.15	1.75–58.97	0.010
Low	74	Reference		
Farmer reported a health problem between 11 and 42 days				
Yes	49	12.47	4.21–36.91	< 0.01
No	211	Reference		
Phone call between the farmer and production technician between 11 and 42 days				
Yes	109	5.87	2.32–14.85	< 0.01
No	151	Reference		

## Discussion

In this study, factors influencing AMU on free-range broiler farms were investigated in a large number of farms representative of the main production areas in France. The results clearly demonstrate that, in addition to technical factors such as housing characteristics, farmers’ perceptions of poultry health play an important role in the use of antimicrobials.

### Study limitations

We recognize that the study may have had some limitations.

The preparation of the study and preliminary contacts with the FOs prior to the study both contributed to the low refusal rate and enabled the authors to set up a case recruitment protocol adapted to each FO. Nevertheless, the efficiency of case recruitment varied depending on the FO. In FOs that did not systematically track antimicrobial treatments, it was difficult to obtain an exhaustive record of treatments. In the FOs where information on treatments was obtained from the referent veterinary practices, some cases were likely overlooked for farms that were followed by a different veterinary practice. Our field experience suggests that this situation is quite rare, and non-exhaustiveness of recruitment might thus have been limited in these FOs. Three of the five FOs that directly provided the information on treated flocks typically consulted their farm technicians at irregular intervals, potentially leading to an increased risk of incomplete recruitment for these FOs. Consequently, it was not possible to compare treatment incidence across FOs in this study. Nevertheless, a systematic assessment of the effective status of the flock (case or control) was performed on-farm thanks to a cross-examination of farm registers, prescriptions, invoices and the remains of drug packaging. Attention should be given in the future to the development of systematic recording systems adapted to each FO (for instance, an online questionnaire), which could help FOs and health advisors to monitor AMU and provide a global picture at the population level.

AMU has been recognized as a complex issue which involves multifactorial determinants. Although a wide range of putative factors were examined in the present study, we cannot dismiss the possibility that some relationships were missed. Cases were scattered across the three regions examined, and no geographical pattern was detected in the dataset. This might be explained by the fact that the syndromes reported by the farmers (colibacillosis, digestive disorders) are known to be endemic and widespread in France. However, further studies are needed to confirm this finding, as previous research in Denmark showed that pig farms with higher AMU were clustered in specific geographical areas [20]. The effect of chicken strain also could not be scrutinized, although it could be hypothesized that strain, which has been found to be associated with first week mortality [21], may play a role in AMU variations. All flocks involved in the study were slow-growing strains, but a variety of strains were observed in the field. This resulted in a lack of statistical power when examining this variable.

### Health problems associated with the use of antimicrobials

We found that antimicrobials were mainly used for therapeutic purposes, contrary to that reported by Hughes et al. [17]. Abnormal flock mortality was the main health problem associated with AMU in this study, contrary to previous studies where necrotic enteritis was the main indication of use [14] followed by respiratory disease and then flock mortality [17]. The effect of the factors highlighted in the present study should thus be interpreted in relation to their potential effect on mortality.

The three antimicrobials most frequently used were similar to those previously reported [14, 17]. With regard to critically important antimicrobials authorized for poultry in France, enrofloxacin was frequently observed in this study (10/61), with a median duration of treatment of 3.5 days and an interquartile range of 2. Most treatments were preceded by an autopsy on birds that had been found dead (7/10), with only two preceded by an antibiogram. The use of antimicrobial testing observed in this study may be considered low in the light of the current recommendations regarding the use of critically important antimicrobials [22]. However, the questionnaire used and the farm documents examined may have only partially captured records of antimicrobial testing. Nevertheless, it is consistent with previous results showing that when prescribing antimicrobials, European veterinarians rely on experience rather than sensitivity testing [23, 24]. Cross-verification with the veterinarian’s records could provide more detailed information on this subject. In addition, a decree published on 2017/03/16 allows the use of critically important antimicrobials only after an autopsy or clinical examination, bacteria isolation and antimicrobial testing. Different results consequently should now be observed.

### Technical preventive factors

First, the use of chicken paper topped with starter feed was associated with a decreased probability of AMU. This technical tool aims to optimize the management of the first 10 days of a chicken’s life. Successful management of this period is crucial for the remainder of the flock’s life and determines its future technical performance, including early mortality [21, 25]. Chicken paper is covered with feed and is set under the drinking and heating systems. The noise produced by chicks walking on the paper attracts the rest of the flock, gathering the birds in the area where all elements needed for their comfort are available. The early satisfaction of their physiological needs ensures the development of their immune system [26], making them less vulnerable to disease and subsequently reduces the need for antimicrobial treatment. Discrepant observations were made by Heier et al. [25], who demonstrated that the use of chicken paper in industrial Norwegian chicken farms was associated with higher mortality. They theorized that the mix of feed and droppings increased infection. These divergent results could be related to the fact that French free-range broiler breeding systems have a lower bird density and less productive chicken strains than conventional Norwegian systems. The amount of time that the chicken paper is left in the poultry house also should be considered.

Second, the use of herbal drugs as a preventive measure was associated with a decreased probability of AMU. The herbal drug treatments recorded in this study were essential oils, and most were administered because they were part of the FO’s prophylactic strategy. Twenty-five out of 37 herbal drug treatments were administered before day 5 and for a median duration of 4 days, with an interquartile range of 5. As each FO has its own prophylactic strategy, the frequency of herbal drug use for prevention varied greatly between the FOs. In our study, it is important to stress that the variable only focused on herbal drugs used for prevention and not to deal with a health problem in the flock. In addition to a potential specific effect of essential oils on disease prevention, it is possible that the association is related to the fact that when farmers adopt herbal drugs, they also engage in a global set of good farming practices. Farmers using herbal drugs tended to implement better management and prevention practices (all in-all out procedure, change of shoes in the hygiene lock, frequent visits to the broilers, acidification of drinking water, use of anticoccidials and Gumboro vaccines) than farmers who did not use herbal drugs. Variables capturing these “good practices” were all tested in the univariable analysis, and significant variables were included in the multivariable analysis. A confounding effect of good practices thus remains limited in the present study. Further experimental studies are needed to assess the effect of herbal drugs, and more specifically of essential oils, in preventing health problems in poultry.

### Farmer perception of poultry health

There was an increased probability of AMU when farmers identified a problem in the flock when the chickens were between 11 and 42 days old. This finding is consistent with the observation that antimicrobial treatments are mostly administered as a therapeutic response to a health problem on the farm: more than 90% of the antimicrobials were administered as a treatment (and not a prophylaxis). The health problems reported by farmers were mainly abnormal mortality (27 out of 72 during the 11–42 day period) (Fig. 4.). Further interpretation of this result is complicated by the lack of additional information regarding the causes of mortality and health issues due to the absence of systematic medical investigations (autopsies, bacterial cultures and sensitivity tests). Mortality is a very common phenomenon in the first week of a chick’s life, and medical investigations are not performed systematically in the field [21, 25, 27–29]. According to the farmers’ declarations, the main causes of mortality were colibacillosis in the first days, and enteritis later on. Olsen et al. [27] showed that among layers dying during their first week, 50% died from infections (mostly omphalitis and yolk sac infection +/− septicemia with a diversity of bacteria that complicates the production of an effective vaccine), and 50% due to non-infectious causes (mostly dehydration and nephropathy). When investigations were performed, little data (autopsy written report, sensitivity testing report) were actually available on farms. These results should be considered in the light of two additional variables. First, phone calls between farmers and technicians were associated with increased AMU. This finding reflects how the FOs operate, with technicians employed by the FO acting as the first-line contact person for farmers when they face a problem in their flock. However, this finding may also reflect farmers’ anxiety and need to be reassured, which could also play a role in AMU. Second, farmer perception of the 10-day mortality was also associated with AMU. Farmer perception of the 10-day mortality rate is compared to the crude objective 10-day mortality rate in Fig. 1. Perceived mortality was preferred for use in the analysis because doubts were raised regarding the robustness and reliability of the data from farm records that were used to calculate the crude objective mortality rate. There were several reasons to doubt the farm records. First, flocks of chicks intended for two different poultry houses were sometimes put in the same poultry house for the first days/weeks of life (to limit energy / heat expenses), with farmers unable to assess the exact number of chicks finally placed in each house. Second, some farmers stated that they had placed some “extra” chicks in a poultry house which were not officially recorded, and they could not give the exact number. The denominator for mortality was thus uncertain. Third, some farmers reported culling the weakest chicks and aggregating culled and dead chicks in farm mortality records. The results of the study thus suggest that the threshold beyond which farmers consider mortality to be abnormal is highly personal. Lupo and Prou [30] studied mortality detection and mortality notification by mussel farmers and assumed that farmers compare their observations to a previous situation when deciding whether to notify. A similar hypothesis can be made here, namely that the way farmers perceive the health status of their flock is partly connected to their farm’s recent health history. The results from the present study suggest that there is a gap between what the farmers perceive as abnormal mortality rate and the actual mortality rate. Often, this perception is highly personal and based on farmers’ experiences with previous flocks, which may influence the AMU in their respective farms. Additional investigation with a different study design could provide complementary information on the discrepancy between objective and perceived mortality. Recent articles [31–34] have emphasized the importance of psycho-social factors (including farmers’ perceptions of health problems and risk associated with antimicrobial resistance, as well as social norms and a belief that it is possible to operate effectively using fewer antimicrobials) in the decision-making process underlying AMU on farms.

### Other factors

The cleaning and disinfection of the concrete perimeter of the poultry house during the previous downtime was associated with an increased probability of AMU. This result was unexpected given that the study was performed during the indoor period when the flock does not have access to the surroundings of the poultry house. It is possible that this finding is a case of reverse causality, with farmers experiencing recurrent health problems trying to eliminate the problem by reinforcing cleaning and disinfecting operations. To better understand this finding, it would have been useful to collect information on the occurrence of health problems in flocks prior to the study period and to directly observe the cleaning and disinfection process (types of molecules used, concentration, duration of application, etc.). Although biosecurity is essential for the control of infectious diseases and thus indirectly impacts antimicrobial usage [11], a questionnaire is not the optimal method to collect data on biosecurity practices [35].

This study also shows that the thinner the litter, the lower the probability of AMU. This result may be seen as counterintuitive as a previous work demonstrated that when the litter is sufficiently thick, broilers are more comfortable (better absorption, better isolation, etc.), and thus less susceptible to diseases [36]. This discrepancy could be explained by differences in the setting, season, or bedding material between the two studies. Other assumptions also could be made to explain our results. First, descriptive statistics suggest that farms that start with less litter have a greater tendency to gradually add litter later (between 11 and 42 days), which could finally improve broiler health and explain lower AMU. In our study, the practice of building up litter thickness over time also was observed more frequently in poultry houses with concrete floors, which can be cleaned and disinfected more efficiently than beaten earth floors. Second, the thickness of the litter could have an indirect negative impact on broiler health. A study showed that the risk of intestinal lesions due to coccidiosis increased with increasing amounts of litter [36], as the broilers have more time to manipulate the litter and subsequently participate in the diffusion and sporulation of oocysts. Further research is needed to investigate the association between litter characteristics, including quantity and type of litter, and AMU.

## Conclusions

In conclusion, this case-control study made it possible to determine the factors associated with AMU during the indoor period of French free-range broiler production systems. Farmers’ perceptions of the health situation, based on their experience and previous flock history, were identified as a major driver of AMU. The use of herbal drugs as a preventive measure was associated with decreased AMU. This highlights the importance of taking into account such innovations in epidemiological studies, and calls for more experimental studies on alternatives to antimicrobials.

## Materials and methods

### Geographic area involved and study period

In France, free-range broilers are mainly produced in the North West and South West regions, which represent 50 and 30% of national production, respectively. Nine farmer organizations (FOs) from these two regions and one FO in central France were contacted for the purpose of the present study; of these, seven ultimately were included (2 North West, 4 South West, 1 Central). These seven FOs encompassed a total of 1930 farmers, which is approximately 38.6% of the total number of farmers involved in the free-range broilers sector in France.

The study covered a period of five months, focusing on flocks placed on farms between 27th November 2015 and 8th April 2016.

### Definition of cases, controls and sample size

A case was defined as a flock of broilers that received at least one antimicrobial treatment between day 1 and 42. All potential cases were recorded during the study period, based on information extracted from the records of the veterinarians working with the farms or from the FO. For each case, controls (i.e., flocks with no antimicrobial treatment between day 1 and 42) were randomly selected from a list of all of the flocks placed by the same FO in a +/− 10-day window around the case placement date. This matching strategy aimed to control confounding bias associated with seasonal climatic effect (for chick placement date) and broad characteristics connected with the FO (such as geographical situation or FO poultry health support strategy) that could not be captured through other variables. Given the low rate of antimicrobial treatments and logistical considerations, the case-control ratio was increased to 1:4 to increase the odds ratio’s precision [37]. The selection of cases was exhaustive. The total sample size, which was set at 315 farms, aimed to detect an odds ratio of 2.5, with 20% of exposed controls, a 5% error and a power of 80%.

### Data collected and questionnaire

The questionnaire included 10 sections (Additional file 1). The first two aimed to gather general data on the farmer and the farm. The third section enabled a random sampling of a poultry house in case the farmer had several poultry houses, and overall insight into the flock history was reported on a historical timeline. The next sections concerned the flock: biosecurity, facilities, hygiene, animal husbandry practices, treatments and prophylaxis. Crude objective mortality rates at 10 and 42 days were calculated based on farm records (cumulative mortality at 10 and 42-days of age divided by total number of chicks placed). Mortality is an indicator of flock performance, and often farmers form their own opinion about the mortality levels in their flocks (based on experience from previous flocks, acceptance/avoidance of stress, etc.). As these perceptions may differ from crude mortality data (as observed during earlier preparatory stages of this study), we collected data on perceived mortality at 10 and 42-days of age (either high, normal or low) in the questionnaire.

The occurrence of health problems was also investigated by asking farmers if, in their opinion, there had been any abnormal event during the period when the chicks were between 1 and 42 days old. When they answered positively, they were asked to describe the type of event they had observed, such as abnormal mortality, digestive disorders, feather pecking, etc. This information was not associated with disease indicators because autopsies and sensitivity tests are not systematically performed when an abnormal event occurs, and even when they are, farmers do not systematically keep the reports. In the absence of objective confirmation of disease occurrence, it was only possible to investigate the farmers’ perception of syndromes that occurred in the flock.

All of the questions were closed questions in French (multiple choice questions, rating scale questions and checklist questions). The questionnaire was pre-tested on three flocks. Three previously trained animal health professionals administered the questionnaires on farms between February and June 2016. The visit, with a mean duration of 1.5 h, included the completion of the questionnaire, the recording and detailed analysis of farm documents (farm register, feed delivery orders, chick delivery orders, and prescriptions), and a visit of the poultry house where the flock being studied was located. At the beginning of the visit, farmers were provided all of the information required regarding their participation in the study to obtain their informed consent.

To limit memory bias, the visit occurred before the broilers were harvested (mean age at harvest in 2015: 86 days [38]). The case-control status of the studied flock was systematically assessed on-farm by examining information on antimicrobial treatments provided both by the questionnaire and farm documents.

### Statistical analysis

The data (previously entered in a Microsoft Access® database) were analyzed using R [39]. Univariable logistic regression, with antimicrobial status (case vs. control) as the binary outcome and the flock as the unit of analysis, was performed to select the candidate variables (*p*-values < 0.25) for multivariable regression analysis. Biologically possible interactions were tested and all potentially connected variables were screened for correlation using a chi-square test. When a strong correlation between explanatory variables was detected (chi-squared test with *p*-value < 0.05), the variables with smaller *p*-values in the univariable analysis and higher biological interest were conserved. Various options exist to account for matching in the statistical analysis of case-control studies [37]. Conditional regression classically is cited, but in the specific case of frequency matching, such as in the present study, it has been recommended to perform standard logistic regression with the matching variable (in this study, the FO) forced as a fixed effect in the final model [40]. Both options were explored in the analysis but standard logistic regression was finally retained. The final model was constructed using a stepwise selection procedure based on Akaike Indicator Criteria. Absence of multicollinearity was assessed (generalized variance inflation factor < 10). The goodness-of-fit of the model was assessed using the Hosmer-Lemeshow test.

## Additional file

Additional file 1: Questionnaire used in the case-control study of risk factors for the use of antimicrobials in French free-range broilers (*n* = 260 flocks) in 2016. (PDF 935 kb)

## Data Availability

The datasets generated and/or analyzed during the current study are available from the corresponding author upon request.

## Abbreviations

AMR: Antimicrobial resistance
AMU: Antimicrobial use
CI: Confidence Interval
FO: Farmer Organizations
IQR: InterQuartile Range
OR: Odds-Ratio
