# Supplementary material for: Epidemiological assessment of the factors associated with antimicrobial use in French free-range broilers
Source: BMC Vet Res. 2019 Jun 28;15:219. doi: 10.1186/s12917-019-1970-1 (PMC6599332; doi:10.1186/s12917-019-1970-1)
Supplement: Supplementary file 1 — Questionnaire used in the case-control study of risk factors for the use of antimicrobials in French free-range broilers (n = 260 flocks) in 2016. (PDF 935 kb) [file 12917_2019_1970_MOESM1_ESM.pdf]

Questionnaire : Farming practices and antimicrobial use in free-range broilers

Farmer's details :

Full name of the farm : |____|

Address : |____|

ZIP Code : |____|

City : |____|

E-mail address : *for any further information or for feedback on the study*
_________________________________@_______________________________

Phone number :
|____|

Farmer organization :
|____|

Part 1: Farm description

The respondant must be the person in charge of the poultry farming activities.

1.1 Name :|____|

1.2 Surname :|____|

1.3 Year of birth *(YYYY)* : |_________________|

1.4 When did you start working on the farm *(YYYY)* ? |_________________|

1.5 What is the highest academic degree you have obtained?
|____|

1.6 Have you received any specialized training in poultry farming ?

☐ Yes

☐ No

1.7 Did you participate to training sessions organized by the farmer organization ? *Several possible answers*

☐ Training / technical sessions organized by the farmer organization

☐ Training / technical sessions organized by a veterinary practice

☐ Visiting other poultry farms

☐ Other : *specify* ____

☐ None

1.8 Do you have any other livestock production, besides poultry ? *Several possible answers*

☐ None

☐ Suckling cattle : ___________ heads

☐ Dairy cattle : ___________ heads

☐ Small ruminants : ___________ heads

☐ Swine : ___________ heads

☐ Other, specify : ___________ heads

1.9 How many people have been working on the farm this year ? ___________________________________

1.10 Apart from specific tasks (cleaning and disinfection, catching, …), how many people are involved in routine in poultry farming ? ___________________________________

1.11 Is there any external workers for poultry farming ?

- Placement of the chicks : ☐ Yes
  ☐ No
- Catching : ☐ Oui
  ☐ No
- Cleaning / disinfection : ☐ Yes
  ☐ No

1.12 How many poultry farms is there in a 3-km radius around your farm ?

☐ None

☐ 1

☐ 2

☐ 3

☐ 4

☐ 5 to 10

☐ More than 10

1.13 What was (in 2015) your average monthly income, including all type of activities?

|____|

1.14 In 2015, which part of your income did free-range broilers farming represent?

☐ Total (100%)

☐ Mostly (75-100%)

☐ Very important (50-75%)

☐ Important (25- 50%)

☐ Secondary or minor (less than 25%)

Part 2 : Poultry production

2.1 Is there any backyard (either on the farm or with neighbors) at a distance of less than 200m from the poultry house :

☐ Yes

☐ No

2.2 Did you raise any other poultry apart from free-range broilers in 2015 ?

☐ No

☐ Yes

2.3 If yes, fill the following table :

		**Total number of flocks placed in 2015**	**Average flock size (no heads)**
**CHICKEN**	Conventional		
	Organic		
	Free-range ➔ **WILL BE DESCRIBED ON THE NEXT SECTION**		
	Other (specify)		
**TURKEY**	Conventional		
	Traditional		
**GUINEA FOWL**	Conventional		
	Free-range		
	Organic		
**DUCKS OR GEESE**	Foie-gras ducks/geese		
	Meat ducks/geese		

2.4 Totally, how many poultry houses do you have on the farm ? |_____________|

The next questions will focus only on free-range broilers

2.5 Since when have you been raising free-range broilers ? (*YYYY*)? |______________|

2.6 When did you start raising free-range broilers ? (*YYYY*)? |______________|

2.7 Did you ever move from one farmer organization to another one ?

☐ Yes

☐ No

Free-range broilers raised in 2015 :

**2.8 Type of poultry house**	**2.9 Area (in m²)**	**2.10 Number of poultry houses**	**2.11 Average size of the flock (no heads)**	**2.12 Total number of flocks placed in 2015**

2.13 Did you produce any flocks under « antibiotic-free » labelling ?

- In 2015 ? Number : |_____________________________|
- In 2016 ? Number : |_____________________________|

Part 3 : Definition of the study flock

The questionnaire will focus on <u>one flock only</u> : if there are several flocks present on farm at the same date, a random sampling is necessary.

<u>CONTROL FLOCK</u> :

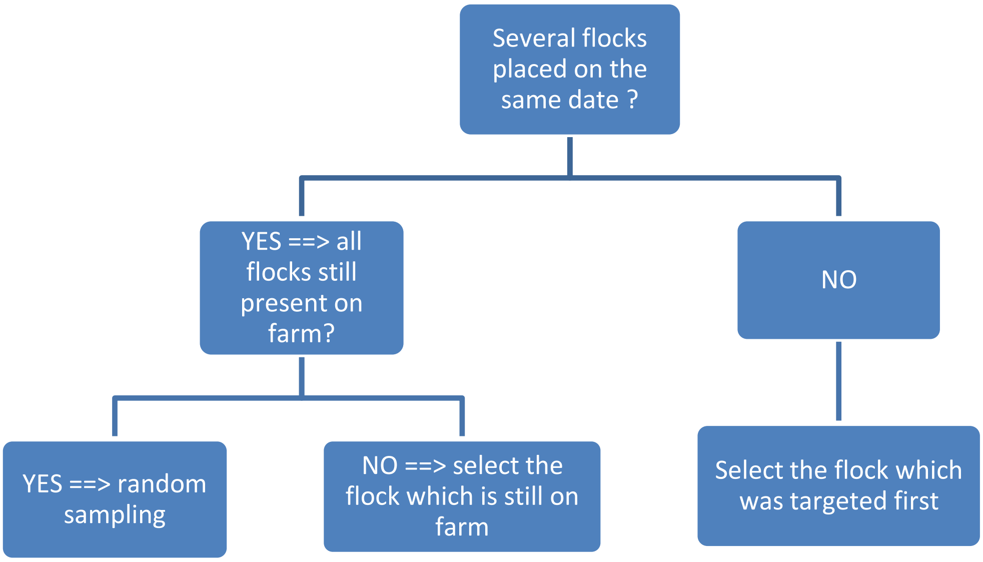

<u>CASE FLOCK</u> :

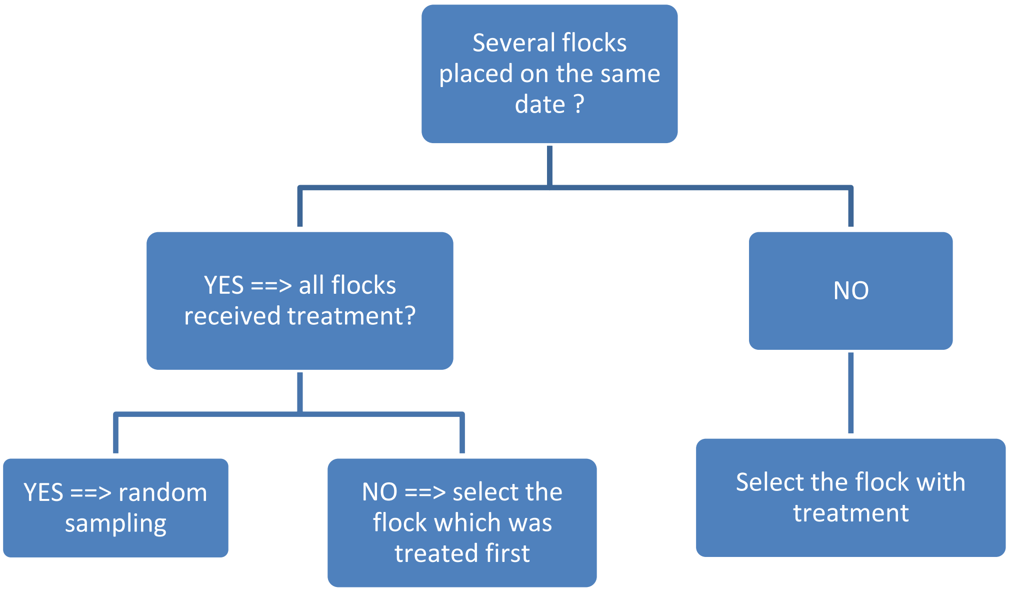

3.1 Date of placement for the study flock : |___________________________|(*DD/MM/YYYY),* noted on the farm register as :

- ☐ **D0**
- ☐ **D1**
- ☐ **D2**

3.2 Identification of the poultry house for the study flock (INUAV code) : V|__________|

Timeline to fill with the farmer (check with the farm register)

List all the treatments used, as well as all abnormal events (mortality, pecking, drop in feed/water consumption, ….)

Health problems

D0 **D10** **D42**

Treatments used

Visit of the technician or veterinarian

Part 4: Biosecurity

4.1 Was there any other poultry species present in other houses when the free-range flock has been placed ?

☐ No (I do not have other poultry house, or the other houses were empty)

☐ Yes ➔ specify species : |____________________________|

4.2 Did you make sure that younger birds are visited before the others ?

☐ Yes

☐ No

4.3 Are carcasses removed by a rendering company ?

☐ Yes

☐ No : specify ___________________________

4.4 Did visitors (friends, family, neighbors) enter the poultry house between 0 and 42 days of age ? (*excluding employees, technicians and veterinarians*)?

☐ No

☐ Yes ➔ number of times : |____________________________|

4.5 Is there any hygiene lock in the poultry house where the flock has been raised ?

☐ Yes

☐ No

4.6 During the period from 1 to 42 days of age, how often did you clean the hygiene lock ?

☐ Once a day

☐ Every other day

☐ Once or twice a week

☐ Every two weeks

☐ Once a month

☐ Never

4.7 How is the hygiene lock organized ?

☐ One zone only

☐ Two zones (clean and dirty), with a physical barrier

☐ Two zones (clean and dirty), with no physical barrier (including lines on the floor)

4.8 During the period from 1 to 42 days of age, did you manage to respect zoning in the hygiene lock ?

☐ Yes, systematically

☐ Mostly during starting (10 first days), less after

☐ Not systematically over the period

☐ No, never

4.9 During the period from 1 to 42 days of age, did you use footbath ?

☐ No

☐ Yes

4.10 If yes, how often did you change footbath during the period from 1 to 42 days of age?

☐ Once a day

☐ Every other day

☐ 2 to 4 times per week

☐ Once a week

☐ Less than once a week

☐ Never

4.11 How did you manage shoes before entering the poultry house (between 1-42 days) ?

☐ Nothing special

☐ I rinsed or changed my shoes ➔ *fill the following table*

☐ **Footbath**	☐ Sytematically, over the period
	☐ Mainly during starting period (1-10 days)
	☐ Not really systematically
☐ **Rinsing shoes**	☐ Sytematically, over the period
	☐ Mainly during starting period (1-10 days)
	☐ Not really systematically
☐ **Changing shoes**	☐ Sytematically, over the period
	☐ Mainly during starting period (1-10 days)
	☐ Not really systematically

4.12 Did you have specific clothes for visiting the poultry house (between 1-42 days) ?

☐ No

☐ Yes, and I used it systematically before moving to the clean area

☐ Yes, and I used it moslty during starting period (1-10 days)

☐ Yes, but I did not use it systematically (only from time to time)

Part 5 : Facilities and equipment

CHARACTERISTICS OF THE POULTRY HOUSE in which the study flock was raised			
**5.1 Type**	**5.2 Surface**	**5.3 Age of the building**	**5.4 Renovation (year)**

5.5 Type of floor

☐ Beaten earth floor

☐ Concrete floor

☐ Other ➔ specify : ____

5.6 Do you have any electronic monitoring equipment in the poultry house ?

☐ No

☐ Yes : name______________________________________

MONITORING EQUIPMENT :

TEMPERATURE	
**5.7 Type of equipment**	**5.8 How often do you check?**
☐ None	
☐ Thermometer or sensor	□ Everyday<br>□ Several times per week<br>□ Once a week<br>□ Mainly during starting (1-10 days)<br>□ Never
☐ Electronic device (connected to sensor)	□ Everyday<br>□ Several times per week<br>□ Once a week<br>□ Mainly during starting (1-10 days)<br>□ Never

WATER CONSUMPTION	
**5.9 Type of equipment**	**5.10 How often do you check ?**
☐ None	
☐ Water meter	□ Everyday<br>□ Several times per week<br>□ Once a week<br>□ Mainly during starting (1-10 days)<br>□ Never
☐ Water level in the tank / metering pump	□ Everyday<br>□ Several times per week<br>□ Once a week<br>□ Mainly during starting (1-10 days)<br>□ Never
☐ Electronic device	□ Everyday<br>□ Several times per week<br>□ Once a week<br>□ Mainly during starting (1-10 days)<br>□ Never

FEED CONSUMPTION	
**5.11 Type of equipment**	**5.12 How often do you check?**
☐ None	
☐ Electronic device	□ Everyday<br>□ Several times per week<br>□ Once a week<br>□ Mainly during starting (1-10 days)<br>□ Never
☐ Other : ____________________	□ Everyday<br>□ Several times per week<br>□ Once a week<br>□ Mainly during starting (1-10 days)<br>□ Never

**POULTRY WEIGHT**	
**5.13 Type of equipment**	**5.14 How often do you check ?**
☐ None	
☐ Manual weighing	□ Everyday<br>□ Several times per week<br>□ Once a week<br>□ Mainly during starting (1-10 days)<br>□ Mainly during growth (11-42 days)<br>□Only for mandatory weighing (organization's instructions)
☐ Electronic weighing	□ Everyday<br>□ Several times per week<br>□ Once a week<br>□ Mainly during starting (1-10 days)<br>□ Mainly during growth (11-42 days)<br>□Only for mandatory weighing (organization's instructions)

**DRINKING**		
**5.15 Type of equipment**	**5.16 Number of equipment during the starting period (1-10 days)**	**5.17 Number of equipment (during growth)**

**FEEDING**		
**5.18 Type of equipment**	**5.19 Number of equipment during the starting period (1-10 days)**	**5.20 Number of equipment (during growth)**

**HEATING**		
**5.21 Type of equipment**	**5.22 Number of equipment during the starting period (1-10 days)**	**5.23 Number of equipment (during growth)**

CLEANING AND DISINFECTING OPERATIONS

5.24 Date of removal of the previous flock (DD/MM/YYYY)? |___________________________|

5.25 How long did cleaning and disinfection last ? |____________________________|

5.26 Did you scrap and sweep the floor after removing the litter from the previous floor ?

☐ **Yes**

☐ No

5.27 Has the building been disinsected ?

☐ No

☐ Yes, just after removing the previous flock

☐ Yes, more than one hour after removing the previous flock

5.28 After removing the previous flock, what did you <u>clean</u> ?

☐ Hygiene lock

☐ Surroundings of the building

☐ Removable material : water troughs, feeders

☐ Floor of the house

☐ Walls of the house

☐ Ceiling (even partially)

☐ Feed silo

5.29 After removing the previous flock, what did you <u>disinfect</u> ?

☐ Hygiene lock

☐ Surroundings of the building

☐ Removable material : water troughs, feeders

☐ Floor of the house

☐ Walls of the house

☐ Ceiling (even partially)

☐ Feed silo

5.30 Was the drinking water system properly cleaned and disinfected before the chicks were put in place?

☐ Yes

☐ No

5.31 Was the litter disinfected before the chicks were put in place?

☐ Yes

☐ No

Part 6 : Management of the starting period (1-10 days)

6.1 Did you use chicken paper toped with starter feed ?

☐ Yes

☐ No

6.2 What thickness of litter has been installed ?

☐ 10 cm or less

☐ 11 to 15 cm

☐ 15 cm or greater

6.3 How many chicks have been found dead at arrival on the farm for the study flock?

☐ I did not count

☐ None

☐ 1 to 5

☐ 5 to 10

☐ More than 10

6.4 Have you checked the chicks ombilic at their arrival ?

☐ No

☐ Yes

6.5 At their arrival,did you assessed their dehydratation ?

☐ No

☐ Yes

6.6 Within 6 hours after their placement, have you checked that the chicks were drinking ?

☐ No

☐ Yes

6.7 Within 6 hours after their placement, have you checked the chicks crop ?

☐ No

☐ Yes

6.8 How many times per day did you visit the poultry house ?

From 1 to 5 days : |____|

From 6 to 10 days : |____|

6.9 From 1 to 10 days, how much time did you spend per day on average in the poultry house for the study flock ? |______________________________| hours

6.10 What was the cumulative mortality rate at 10 days ? |__________| %

6.11 Did you perceive this rate as :

- ☐ Low
- ☐ Normal
- ☐ High

Part 7 : Management of the growth period (11 – 42 days)

7.1 Did you add litter in the poultry house between 11 to 42 days ?

☐ Yes

☐ No

7.2 How many times per day did you visit the poultry house from 11 to 42 days ?
|__________________________|

7.3 From 11 to 42 days, how much time did you spend per day on average in the poultry house for the study flock ? |_____________________________| hours

7.4 Did you eliminate sick or weak birds ?

☐ Yes, systematically

☐ No

7.5 What was the cumulative mortality rate at 42 days ? |_________| %

7.6 Did you perceive this rate as :

☐ Low

☐ Normal

☐ High

7.7 Did you check the litter quality everyday?

☐ Yes

☐ No

Part 8 : Technical support

8.1 Name of the technician in charge of the study flock :

|____|

8.2 Interactions with the technician regarding the study flock :

	**0 – 10 days**	**11 – 42 days**
**8.3 Number of phone calls**		
**8.4 Number of visits on farm**		

8.5 Name(s) of the veterinarian(s) in charge of the study flock :

|____|

|____|

8.6 Interactions with the veterinarian regarding the study flock :

	**0 – 10 days**	**11 – 42 days**
**8.7 Number of phone calls**		
**8.8 Number of visits on farm**		

Part 9 : Farm inputs

FEED

9.1 Was there any probiotics, prebiotics or gut flora stablisers in the feed ?

☐ Yes

☐ No

9.2 Was there any coccidiostats, anticoccidials or ionophores in the feed ?

☐ Yes

☐ No

DRINKING WATER

9.3 What is the source of the drinking water ?

☐ Surface water

☐ Network

☐ Water well

9.4 Was the quality of the drinking water checked last year ?

☐ No

☐ Yes

9.5 Was pH checked in the water analysis ?

☐ Don’t know

☐ No

☐ Yes ➔ pH : |____|

9.6 Was a bacteriological analysis carried out ?

☐ Don’ know

☐ No

☐ Yes, everything was fine

☐ Yes, and a problem was detected ➔ specify :

|____|

9.7 Was drinking water acidified during the first 42 days ?

- ☐ No
- ☐ Yes, continuously after 10 days
- ☐ Yes, during the dietary transition
- ☐ Other : specify |____|

CHICKS

9.8 Where did did the chicks come from (name of the hatchery) ?

|____|

9.9 What was the skin phenotype of the study flock ?

- ☐ White
- ☐ Yellow
- ☐ Black

9.10 What was the name of the strain of the study flock ?

|____|

9.11 Was a coccidia vaccine performed at the hatchery ?

- ☐ No
- ☐ Yes : name |____|

9.12 List of all the vaccines performed at the hatchery :

____

____

____

9.13 List all the vaccines performed by the farmer for the study flock :

Name of the vaccine	Date of vaccination

Part 10 : Health problems and treatments

HEALTH PROBLEM 1 (see the timeline and check the farm register)

10.1 Age of the birds at the beginning of the health problem : D______

10.2 Duration of the problem : _______ days

10.3 What type of problem has been noticed? *Several possible answers*

- ☐ Mortality
- ☐ Pecking
- ☐ Dehydration
- ☐ Omphalitis
- ☐ Digestive disorder
- ☐ Respiratory disorder
- ☐ Leg disorder
- ☐ Other (specify) : ____

10.4 What other signs did you notice ? (including birds' behavior)

____

____

10.5 Did you notice a drop in water consumption ?

- ☐ Yes
- ☐ No / I can not evaluate

10.6 Did you notice a drop in feed consumption ?

- ☐ Yes
- ☐ No / I can not evaluate

10.7 Did you notice any change regarding the litter ?

- ☐ Yes : specify _________________________________
- ☐ No

10.8 Did you notice any change in the broilers' growth ?

☐ Yes

☐ No / I can not evaluate

10.9 Did the technician come to the see the flock for this health problem ?

☐ No

☐ Yes : date of the visit |____|

10.10 Did the veterinarian come to the see the flock for this health problem ?

☐ No

☐ Yes : date of the visit |____|

10.11 Which type of analysis have been made ?

☐ None

☐ Autopsy

☐ Bacteria culture tests

☐ Antibiogram

☐ Other : Specify |____|

10.12 Was there any treatment implemented in response to this health problem ?

☐ No

☐ Yes ➔ *Provide more details*

HEALTH PROBLEM 1, TREATMENT 1

T.1.1.1 Brand name : |____|

T.1.1.2 Date of beginning of the treatment : |____|

T.1.1.3 Duration : |____|

T.1.1.4 Who initiated the treatment ?

☐ Yourself (you had leftovers or you have gone to buy the product)

☐ The technician

☐ The veterinarian

T.1.1.8 Did you follow the prescription ?

☐ Yes

☐ No, I shortened the treatment as birds were going better

☐ No, I extended the treatment

TREATMENTS USED IN THE ABSENCE OF HEALTH PROBLEM

Prophylactic product 1

P.1.1 Brand name : |____|

P1.1.2 Date of beginning of the treatment : |____|

P1.1.3 Duration : |____|

PT.1.1.4 Who initiated the treatment ?

- ☐ Yourself (you had leftovers or you have gone to buy the product)
- ☐ The technician
- ☐ The veterinarian
- ☐ Another farmer

P.1.1.5 For what purpose did you use this product ?

____

____

Additional comments
